# Chlorophyll and Carotenoid Metabolism Varies with Growth Temperatures among Tea Genotypes with Different Leaf Colors in *Camellia sinensis*

**DOI:** 10.3390/ijms251910772

**Published:** 2024-10-07

**Authors:** Pengfei Xu, Jingbo Yu, Ruihong Ma, Yanyan Ji, Qiang Hu, Yihu Mao, Changqing Ding, Zhengzhen Li, Shibei Ge, Wei-Wei Deng, Xin Li

**Affiliations:** 1 Key Laboratory of Tea Quality and Safety Control, Ministry of Agriculture and Rural Affairs, Tea Research Institute, Chinese Academy of Agricultural Sciences, Hangzhou 310008, China; xpfelements@163.com (P.X.); jingboyu625@163.com (J.Y.); 17863935460@163.com (R.M.); jecho1998@163.com (Y.J.); huqiang@tricaas.com (Q.H.); maoyihu@tricaas.com (Y.M.); chqding@tricaas.com (C.D.); lizhengzhen@tricaas.com (Z.L.); geshibei@tricaas.com (S.G.); 2 State Key Laboratory of Tea Plant Biology and Utilization, Anhui Agricultural University, Hefei 230036, China

**Keywords:** albino tea, cold, green tea, high temperature, photosynthetic pigment

## Abstract

The phenotype of albino tea plants (ATPs) is significantly influenced by temperature regimes and light conditions, which alter certain components of the tea leaves leading to corresponding phenotypic changes. However, the regulatory mechanism of temperature-dependent changes in photosynthetic pigment contents and the resultant leaf colors remain unclear. Here, we examined the chloroplast microstructure, shoot phenotype, photosynthetic pigment content, and the expression of pigment synthesis-related genes in three tea genotypes with different leaf colors under different temperature conditions. The electron microscopy results revealed that all varieties experienced the most severe chloroplast damage at 15 °C, particularly in albino cultivar Baiye 1 (BY), where chloroplast basal lamellae were loosely arranged, and some chloroplasts were even empty. In contrast, the chloroplast basal lamellae at 35 °C and 25 °C were neatly arranged and well-developed, outperforming those observed at 20 °C and 15 °C. Chlorophyll and carotenoid measurements revealed a significant reduction in chlorophyll content under low temperature treatment, peaking at ambient temperature followed by high temperatures. Interestingly, BY showed remarkable tolerance to high temperatures, maintaining relatively high chlorophyll content, indicating its sensitivity primarily to low temperatures. Furthermore, the trends in gene expression related to chlorophyll and carotenoid metabolism were largely consistent with the pigment content. Correlation analysis identified key genes responsible for temperature-induced changes in these pigments, suggesting that changes in their expression likely contribute to temperature-dependent leaf color variations.

## 1. Introduction

Tea [*Camellia sinensis* (L.) Kuntze], an evergreen perennial crop valued for its unique taste and rich nutritional content, has become one of the most popular beverages worldwide [1]. Tea plants have a long domestication history that is traced back to 3000 years ago [2]. Throughout long evolution, the evergreen characteristic of tea plants has undergone mutations, leading to a phenomenon known as the albino phenotype (also referred to as albinism) [3]. Albinism, in which the leaf becomes white, is common in many plants, such as Arabidopsis (*Arabidopsis thaliana*), Tomato (*Solanum lycopersicum*), Maize (*Zea mays*), Rice (*Oryza sativa*), etc. [4,5,6,7]. For most crops, albinism has adverse effects, leading to reduced yields and deteriorated quality due to damage to photosynthetic organs, resulting in an imbalance in carbon and nitrogen metabolism [8]. Compared with normal green tea plants, the L-theanine content in Albino tea plants (ATPs) is 0.5–2 times higher, while the total catechin content is only half that of normal tea plants [9]. ATPs possess a unique ratio of polyphenols to amino acids, enhancing the freshness while simultaneously reducing the bitterness of the tea [10,11]. Thus, tea leaf color variation resources serve as essential materials for genetic breeding programs and represent a crucial source for the development of high value-added products.

In China, ATPs are becoming increasing attention, and their planting range is gradually expanding, with a gradual introduction from low altitude to high altitude. Various ecological factors, including light, temperature, humidity, rainfall, and soil conditions, affect the flavor and aroma of tea [12,13,14]. Notably, temperature not only has a great impact on the quality-related substances of the tea but also on the pigment content of the tea leaves, which should not be overlooked. Zhao et al. found that chlorophyll content decreases under low temperature conditions due to the inhibition of chlorophyll biosynthesis enzymes [15]. Carotenoid biosynthesis is regulated by various factors, including environmental conditions, endogenous signals, and transcriptional levels of biosynthetic genes [16,17,18]. ATPs can be classified according to the different environmental conditions of albinism: temperature sensitive, light sensitive and ecologically sensitive. In temperature-sensitive tea plants, the new buds typically sprout and turn white when temperatures are below 20 °C with the upper threshold between 20 and 22 °C, and when the temperature is greater than 23 °C, the phenomenon of whitening does not occur. On the other hand, the start-up temperature of re-greening is about 16–18 °C, and at a temperature lower than 16 °C, the re-greening speed is slowed down [19]. Light and temperature can regulate the accumulation of secondary metabolites at multiple levels, particularly, environmental factors at different temperatures affect polyphenol accumulation [20].

Differential expression of genes related to the chlorophyll and carotenoid metabolic pathways results in alterations in the relative amounts of these two photosynthetic pigments, leading to albino phenotype in ‘Baiye 1’ (BY) and ‘Zhonghuang 1’ (ZH) tea cultivars [21,22]. The typical chlorophyll synthesis pathway involves the sequential synthesis of the following metabolites: glutamate (Glu) → 5-aminolevulinic acid (ALA) → protoporphyrin IX (Proto IX) → magnesium protoporphyrin IX (Mg- Proto IX) → chlorophyll a (Chl a) → chlorophyll b (Chl b). Mutations in any of the 16 genes involved in the chlorophyll biosynthesis pathway may lead to changes in leaf color in plants. Expression analysis of chlorophyll synthesis-related genes showed that transcript levels of the glutamate-tRNA reductase gene (*CsGluTR*), chlorophyll synthase gene (*CsChl*), and chlorophyllate acetate oxidase gene (*CsCAO*) greatly differ at different stages of albinism in ‘Baiye 1’, suggesting that the blockage in the chlorophyll synthesis pathway is the direct cause of albino phenotype of ‘Baiye 1’ [23]. A comparative study of photosensitive albino tea ‘Huangjinya’ and normal green leaf tea ‘Fudingdabai’ revealed that strong light regulated the expression of carotenoid synthesis gene-related pathways, leading to the accumulation of carotenoids, and that the lack of chlorophyll and the accumulation of carotenoids were responsible for the yellow coloration or albino phenotype of new shoots in ‘Huangjinya’ [18]. The expression level of lycopene β-cyclase gene (*CsLCYb*), a key gene for carotenoid synthesis in tea plants, was positively correlated with the degree of albinism in different leaf positions of the albino variety ‘Zhonghuang 2’ and was consistent with the level of carotenoid content. It was also found that the relative expression of this gene was higher in ‘Zhonghuang 2’ than in ‘Longjing 43’ (LJ) [24].

The current study aims to investigate the impact of different temperature conditions on leaf color phenotype, photosynthetic pigment content, and associated gene expression in three tea cultivars differing in leaf colors. It is important to note that different tea varieties have varying tolerance and response to environmental factors, indicating their unique response mechanisms. Therefore, it is necessary to study the effect of temperature on the leaf color phenotype of different tea varieties and the response mechanism of tea plants with different leaf colors to multiple temperature conditions. This study can provide a theoretical basis for understanding the response mechanism of tea plants to temperature.

## 2. Results and Discussion

### 2.1. Variations in Shoot Phenotype under Different Temperatures

**Figure 1:**
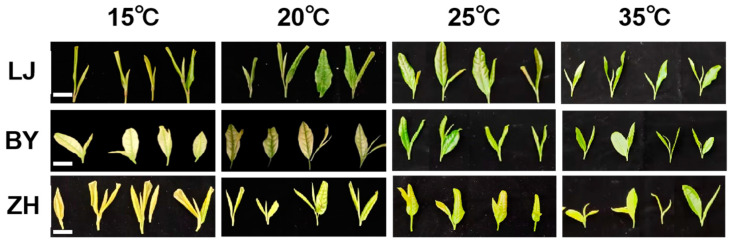
The shoot phenotype (one bud and two leaves) of ‘Longjing 43’ (LJ), ‘Baiye 1’ (BY), and ‘Zhonghuang 1’ (ZH) tea cultivars under 15 °C, 20 °C, 25 °C, and 35 °C temperature conditions.

Under different temperature treatments, the shoot color of ‘BY’ gradually changed from white to green as the temperature increased (Figure 1). Similarly, the shoot color of the albino cultivar ‘ZH’ shifted from yellow to green with rising temperatures, although the extent of this change was less pronounced compared to ‘BY’. In contrast, ‘LJ’ exhibited minimal color change, maintaining its evergreen characteristic across the temperature variations.

Such differences in leaf color changes may be attributed to the diverse responses of certain metabolic pathways to temperature changes in the albino tea plants. Previous studies on the albino cultivar ‘BY’ have established its critical temperature threshold, with leaves turning white at temperatures below 20 °C, and our findings corroborate this phenomenon [21,25]. In the case of ‘ZH’, the observed changes were less significant. Wang et al. found that both ‘ZH1’ and ‘ZH2’ were influenced by both temperature and light, with single temperature changes having a lesser impact on leaf color [26].

### 2.2. Effects of Different Temperatures on Chloroplast Ultrastructure

**Figure 2:**
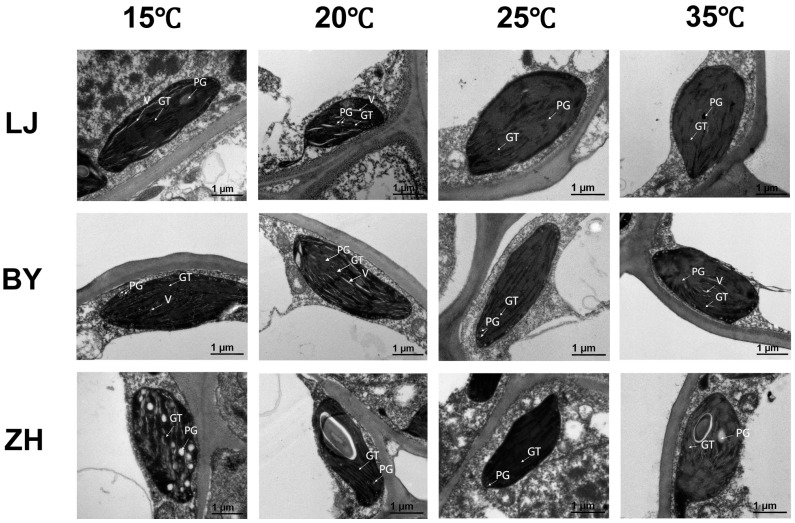
The changes in chloroplast ultrastructure of ‘Longjing 43’ (LJ), ‘Baiye 1’ (BY), and ‘Zhonghuang 1’ (ZH) tea cultivars under 15 °C, 20 °C, 25 °C, and 35 °C temperature conditions. The black ellipse is the chloroplast; the white area within the ellipse is the gap caused by poorly aligned chloroplast basal granules; and the gray stripe is the cell wall. GT, grana thylakoid; PG, plastoglobule; V, void. All photographs are scaled to 1 μm.

Chloroplasts, specialized plastids in higher plants and algal cells, perform photosynthesis and are crucial in the synthesis of pigments, amino acids, and fatty acids [27]. The size and number of chloroplasts in higher plants often vary across species, but their structure is relatively stable, typically oval with a smooth surface. Some have small vesicles protruding from the outer membrane. The inner basal lamellae of chloroplasts are tightly stacked, neatly arranged, and structurally intact. Chloroplasts can rapidly respond to environmental changes, and their development can be influenced by various factors, such as temperature [28]. Low temperatures often damage the morphology, structure, and function of chloroplasts [29,30]. Transmission electron microscopy (Figure 2) has shown that at 25 °C, chloroplasts in the BY are intact and the basal lamellae are tightly arranged. However, at 15 °C and 20 °C, the arrangement of chloroplast basal lamellae is much looser. Particularly at 15 °C, some chloroplasts appear empty. LJ and ZH also exhibit similar trends to varying degrees, though not as pronounced as in ‘BY’. At higher temperatures, chloroplasts in these three tea varieties appear normal. These results indicate that low temperatures, rather than high temperatures, disrupt the chloroplasts in tea leaves, causing developmental abnormalities. Among the three varieties, BY is the most sensitive to low-temperature treatment, showing the most severe damage at these conditions.

### 2.3. Changes in Photosynthetic Pigment Content under Different Temperatures

**Figure 3:**
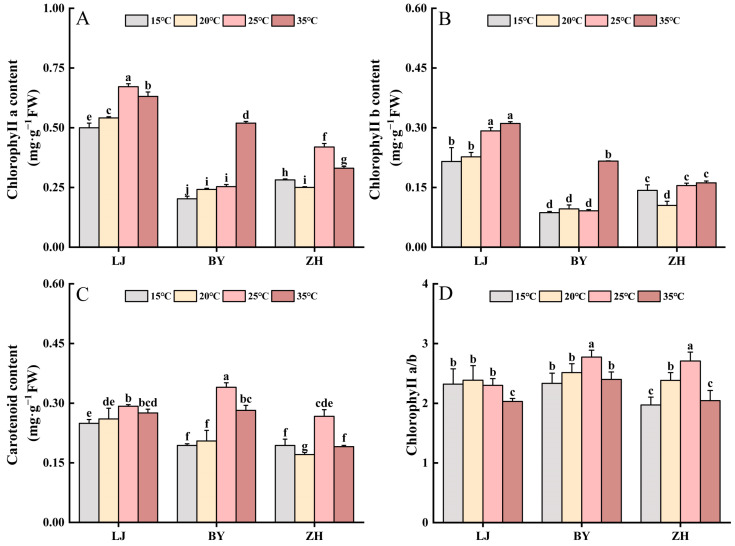
Effects of different temperature conditions on photosynthetic pigment content. (**A**) Chlorophyll a content. (**B**) Chlorophyll b content. (**C**) Carotenoid content. (**D**) Chlorophyll a Chlorophyll b ratio. FW, fresh weight (each bar shows the mean, n = 3). Lower case letters represent the level of significance (*p* < 0.05) between treatment groups. Groups with non-repeated letters are statistically significant, while groups with the same letter are not significantly different from each other.

In the four temperature treatments, the descending sort of chlorophyll a content in leaves of BY was 35 °C, 25 °C, 20 °C, and 15 °C (Figure 3A,B). However, the highest chlorophyll a content in leaves of LJ and ZH was when treated with 25 °C (Figure 3A,B). The chlorophyll b content in leaves of LJ, ZH, and BY decreased with the decreasing temperature. These results showed that BY exhibited chlorophyll content reduction only at low temperatures, whereas the chlorophyll content of LJ and ZH was affected by both high and low temperatures. This finding is consistent with the previous results that BY is a low-temperature-sensitive albino tea plant, when the temperature is < 20 °C, the leaf color is white and when the temperature increases to room temperature, the leaves return to a normal green color [31,32]. The descending sort of carotenoid content in leaves of LJ, ZH, and BY was 25 °C, 35 °C, 20 °C, and 15 °C (Figure 3C). Low temperature had a greater effect on the carotenoid content of the three tea plant cultivars, but because BY is a temperature-sensitive albino tea plant, the effect of low temperature on its carotenoid content was greater than that of LJ and ZH. This finding is consistent with the research by Ritonga et al., who demonstrated that photosynthetic pigment content is significantly reduced under cold stress conditions [33]. This is likely to be caused by the suppression of carotenoid synthesis-related genes at low temperature. The ratio of Chlorophyll a/b was suppressed to different degrees under high and low temperature (Figure 3D). Deng et al. investigated the expression of carotenoid-related genes in BY under 15 °C, 19 °C, and 23 °C treatments, and found that the expression of *PSY, LCYB, LCYE*, and *VDE* was suppressed under 15 °C conditions [34]. Du et al. found that the carotenoid content in the leaves of the temperature-sensitive albino tea ‘Xiaoxueya’ was significantly lower at 15 °C than at 25 °C [35]. In the green tea species, low temperature inhibited the accumulation of carotenoids, and the decrease in the carotenoid content was smaller at 15 °C due to the suppression of the expression of the VDE gene at low temperatures [35]. Low temperatures have a greater effect on pigment content than high temperatures [36].

### 2.4. Changes in the Expression of Genes Related to Chlorophyll and Carotenoid Metabolic Pathways

Chlorophyll synthesis-related genes (*PBGS, PBGD*, and *CAO*) play key roles in the biosynthesis of tetrapyrroles and chlorophylls, regulating the production of chlorophyll precursors and the conversion of chlorophyll a to chlorophyll b, which are crucial for photosynthesis and plant adaptation to changing light environments [37,38,39]. The expression levels of chlorophyll synthesis-related genes in BY and ZH were higher than LJ under the four different temperature conditions. This finding aligns with more recent research highlighting the essential roles of *PBGS* and *PBGD* in the tetrapyrrole biosynthesis pathway [37,38]. Similarly, *CAO*, which converts chlorophyllide a to chlorophyllide b, remains vital for maintaining the chlorophyll a/b ratio, ensuring efficient photosynthesis and adaptation to changing light conditions [39].

When compared to LJ, BY and ZH exhibited lower expression levels of genes directly responsible for the synthesis of chlorophyll a and b, particularly *CHLG*, a gene critical in the final steps of chlorophyll biosynthesis. Recent studies confirm that downregulation of *CHLG* results in reduced chlorophyll levels, leading to compromised photosynthetic efficiency [40]. Additionally, BY and ZH showed higher expression levels of chlorophyll degradation-related genes, which could further explain their lower chlorophyll content compared to LJ. This is consistent with recent findings indicating that enhanced expression of chlorophyllase and *PAO* correlates with accelerated chlorophyll breakdown under environmental stress [41].

**Figure 4:**
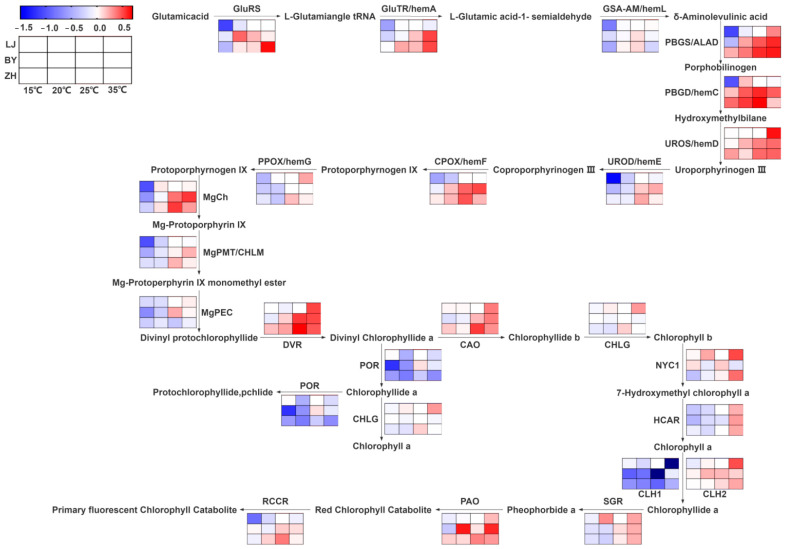
Chlorophyll metabolic pathways and expression levels of chlorophyll metabolism-related genes in LJ, BY, and ZH under 15 °C, 20 °C, 25 °C, and 35 °C temperature conditions.

The expression patterns of most genes in this study corresponded with the trends in chlorophyll content, following the order: 25 °C > 35 °C > 20 °C > 15 °C (Figure 4). These results are in line with studies showing that chlorophyll accumulation typically peaks at moderate temperatures, where photosynthetic machinery operates optimally [42]. Moreover, under the same temperature conditions, the expression levels of *PPOX, MgPEC, CAO*, and *POR* genes in LJ were higher than those in BY and ZH, reflecting the higher chlorophyll content in LJ. The importance of these genes in chlorophyll biosynthesis, particularly under varying environmental conditions, has been revealed by recent studies [43,44,45,46]. Changes in the expression of the *POR* gene are a key factor in triggering albinism in tea plants. Studies have consistently shown that significant reductions in chlorophyll content in albino leaves are linked to downregulated POR expression at the protein level and lower transcripts of associated coding genes [47]. Furthermore, similar observations have been made in other species, such as rice, where the deletion of a guanine base in the second exon of the *PORB* gene in the rice leaf color mutant *fgl* led to a yellowish-green phenotype [48].

**Figure 5:**
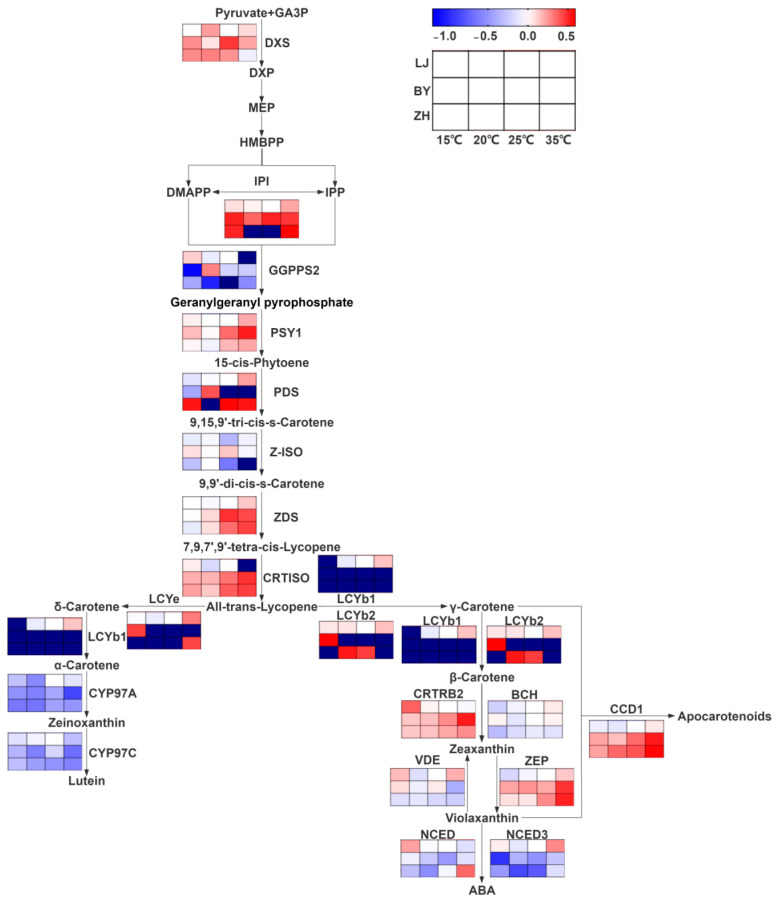
Carotenoid metabolic pathways and expression levels of carotenoid metabolism-related genes in LJ, BY, and ZH under 15 °C, 20 °C, 25 °C and 35 °C temperature conditions.

In addition, the expression trends of most carotenoid synthesis-related genes closely mirrored the trends in carotenoid content (Figure 5). This is consistent with recent studies that emphasize the coordinated regulation of chlorophyll and carotenoid pathways, ensuring a balance between these pigments, which is essential for effective light harvesting and photoprotection [49].

### 2.5. Correlation Analysis of Chlorophyll Metabolism-Related Gene Expression and Chlorophyll Content

**Figure 6:**
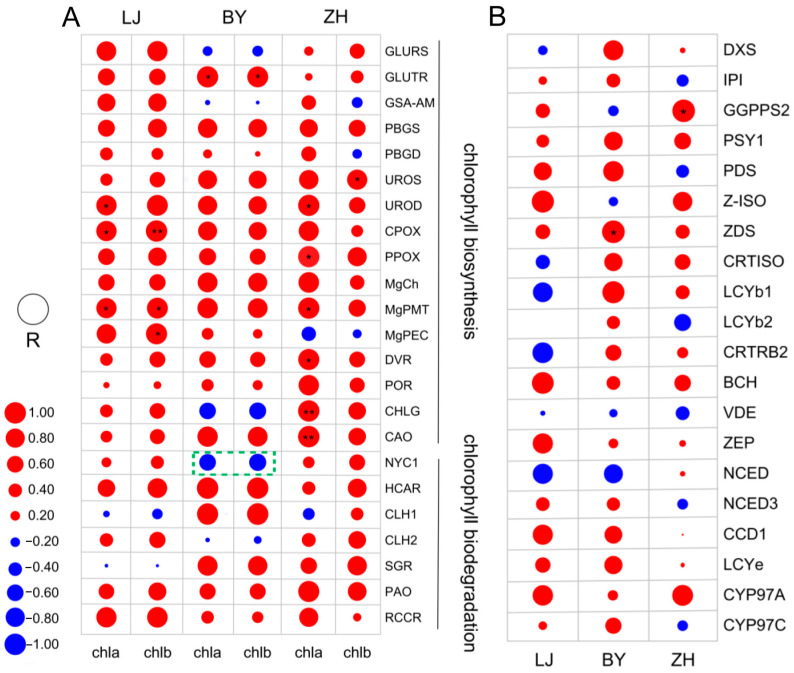
Pearson correlation analysis. (**A**) Chlorophyll content and expression of genes related to chlorophyll metabolism. (**B**) Pearson correlation analysis of carotenoid content and expression of genes related to carotenoid metabolism. Correlation expressed as R-value, with |R| ≥ 0.6 being moderately correlated and |R| ≥ 0.8 being highly correlated (the size of the circle shows the absolute value of R, red represents positive correlation, blue represents negative correlation, and * shows the level of significance: *, *p* < 0.05; **, *p* < 0.01).

Pearson correlation analysis was conducted to examine the relationship between chlorophyll a and b content and the expression of genes involved in the chlorophyll metabolic pathway in LJ, BY, and ZH (Figure 6A). The results revealed that most gene expressions were highly positively correlated with chlorophyll a and b content across different temperatures (Supplementary Table S1). Specifically, *UROD*, *CPOX*, and *MgPMT* expression levels showed a significant positive correlation with chlorophyll a content, while *MgPMT*, *CPOX*, and *MgPEC* expressions were significantly positively correlated with chlorophyll b content (*p* < 0.05). Similarly, in BY and ZH, the expression of most chlorophyll metabolic genes was positively correlated with chlorophyll a and b content. In BY, *GluTR*/*hemA* demonstrated a significant positive correlation with both chlorophyll a and b content (*p* < 0.05). *GluTR*, encoded by *hemA*, is a key regulator of chlorophyll synthesis, catalyzing the formation of ALA from l-glutamyl tRNA, a critical step in chlorophyll biosynthesis [50]. Recent studies on rice chlorophyll-deficient mutants have also identified that mutations in *hemA*, the gene encoding *GluTR*, can lead to a complete blockage of the chlorophyll synthesis pathway, resulting in altered leaf morphology [51].

In addition, *NYC1*, a crucial gene promoting chlorophyll degradation, showed a moderate but not significant negative correlation with chlorophyll a and b content, which is consistent with previous findings [52,53]. In ZH, *UROD*, *PPOX*, *MgPMT*, *CHLG*, and *CAO* expression levels exhibited a highly positive correlation with chlorophyll a content, with *UROD*, *PPOX*, and *MgPMT* showing significant correlations (*p* < 0.05). Recent studies have indicated that under low-temperature stress, *UROD* content decreases significantly, which can impact chlorophyll production [54]. *PPOX*, which catalyzes the oxidation of protoporphyrinogen IX to protoporphyrin IX, is the last shared step in the biosynthesis of both chlorophyll and heme, and its activity directly affects chlorophyll content [55]. In Arabidopsis, knockout mutations of the *CHLM* gene, responsible for magnesium-protoporphyrin IX methyltransferase activity, demonstrated its essential role in chlorophyll formation and the assembly of the photosystem complex [56].

The correlation between *CHLG* and *CAO* expression and chlorophyll a content was highly significant. In the final step of chlorophyll biosynthesis, *CHLG* esterifies chlorophyll a and b with phytol or geranylgeranyl pyrophosphate in the chloroplasts [57]. Furthermore, in ZH, *UROS* expression exhibited a significant positive correlation with chlorophyll b content (*p* < 0.05). These key genes, which show positive and highly significant correlations with chlorophyll content, likely contribute to variations in chlorophyll levels as their expression changes with temperature. Our analysis suggests that the differences in leaf color observed in LJ, BY, and ZH under varying temperatures are more likely due to differential chlorophyll biosynthesis rather than chlorophyll degradation.

### 2.6. Correlation Analysis of Carotenoids Metabolism-Related Gene Expression and Carotenoids Content

To investigate the relationship between the expression of carotenoid pathway-related genes and carotenoid content, we performed Pearson correlation analyses (Supplementary Table S2). In LJ, the expression levels of *Z-IOS*, *BCH*, *ZEP*, *CCD1*, and *CYP97A* exhibited positive correlations with carotenoid content (Figure 6B). This aligns with previous research indicating that *CYP97A*, a cytochrome P450 enzyme, plays a key role in the hydroxylation of carotenoids, impacting carotenoid composition and content in plants [58]. Similarly, *CCD1*, a carotenoid cleavage dioxygenase, has been shown to influence carotenoid degradation and ,thus, affecting overall carotenoid levels [59].

In BY, *DXS*, *PDS*, *ZDS*, and *LCYb1* were highly correlated with carotenoid content, with the correlation between *ZDS* expression and carotenoid content showing significant correlations (*p* < 0.05). This finding is consistent with studies highlighting the importance of *ZDS* (z-carotene desaturase) and *DXS* (1-deoxy-D-xylulose-5-phosphate synthase) in carotenoid biosynthesis, where they serve as critical enzymes in the early steps of the pathway [60,61]. Additionally, *LCYb1* has been identified as a major determinant of carotenoid accumulation, as it controls the cyclization of lycopene to produce β-carotene, a precursor for various other carotenoids [62].

In ZH, the expression of *GGPPS2* and *CYP97A* showed strong positive correlations with carotenoid content, with *GGPPS2* showing significant correlations (*p* < 0.05). This is in line with recent research indicating that *GGPPS2* is crucial for the production of geranylgeranyl diphosphate, a key precursor for carotenoid biosynthesis, and its regulation is vital for carotenoid accumulation [63]. Studies have also emphasized that *GGPPS2* expression is tightly linked to environmental factors, further supporting its role in modulating carotenoid content under varying conditions [64].

Our study clarifies the importance of these genes in regulating carotenoid biosynthesis and highlights the influence of environmental conditions on their expression and, thus, contributing to the observed variation in carotenoid content among the tea plant varieties.

## 3. Materials and Methods

### 3.1. Plant Materials and Treatments

The tea cultivar ‘Baiye 1’ (BY), ‘Longjin43’ (LJ) and ‘Zhonghuang 1’ (ZH) were collected in July 2023 at the Tea Research Institute of the Chinese Academy of Agricultural Sciences (TRI, CAAS, N30°10′, E120°5′). ‘Longjing 43’ is a widely cultivated green-leaf tea variety known for its stress resistance (cold, drought, heat, and heavy metal) and high-quality tea production [65,66,67,68]. ‘Baiye 1’ and ‘Zhonghuang 1’ are temperature-sensitive albino tea varieties, exhibiting white and yellow leaf colors under low temperature conditions, respectively. All tea plants were not obtained through EMS (ethyl methanesulfonate) mutagenesis but were instead bred through natural variation and long-term selection. Plants were set in plastic containers, which were placed within different plant growth chambers with 600 μmol·m^−2^·s^−1^ PAR, 16 h/8 h photoperiod, and 65% relative humidity. All plants were divided into four treatments until the one bud and two leaves stage as follows: (1) Low temperature (15 °C): plants grew at a temperature of 15/10 °C (day/night). (2) Sub-low temperature (20 °C): plants grew at a temperature of 20/15 °C (day/night). (3) Normal temperature (25 °C): plants grew at a temperature of 25/20 °C (day/night). (4) High temperature (35 °C): plants grew at a temperature of 35/30 °C (day/night). Each treatment comprised three biological replicates and was carried out under a completely randomized design (CRD).

### 3.2. Determination of Photosynthetic Pigment Contents

Fresh leaf tissues (0.2 g) were ground into powder and then soaked in 10 mL of 80% acetone and stored in a dark place until it turned white. The absorbance of each sample at 663 nm, 645 nm, and 445.5 nm was determined after zeroing with 80% acetone by an ultraviolet-visible spectrophotometer (UV-6100, METASH, Shanghai, China). Chl content was calculated using the formula illustrated as follows:

Chla (μg/mL) = 9.78 × A_663_ − 0.99 × A_645_

Chlb (μg/mL) = 21.4 × A_645_ − 4.65 × A_663_

Car (μg/mL) = 4.69 × A_445.5_ − (chla + chlb) × 0.268

### 3.3. Transmission Electron Microscopy (TEM) Observation

The fresh leaf samples were fixed with 2.5% glutaraldehyde at 4 °C overnight and rinsed with 0.1 M phosphate buffer (pH 7.0) three times for 15 min each time. The samples were post-fixed with 1% osmium tetroxide for 1–2 h and then washed with 0.1 M phosphate buffer (pH 7.0) three times for 15 min each time. After that, the samples were dehydrated with graded concentrations (30%, 50%, 70%, 80%, *v*/*v*) of ethanol, then dehydrated twice with 100% acetone for 20 min. The dehydrated samples were embedded with a mixture of Spurr resin and acetone 1:1(*v*/*v*) for 1 h, then transferred in a mixture of Spurr resin and acetone (3:1, *v*/*v*) for 3 h. Finally, samples were treated with pure Spurr resin overnight at room temperature. The samples were cut into 70–90 nm sections by using a LEICA EM UC7 ultrathome (Leica, Wetzlar, Germany), and the sections were stained with a uranyl acetate and alkaline lead citrate for 7 min each. Finally, the samples were observed through a Hitachi H-7650 TEM (Hitachi, Tokyo, Japan).

### 3.4. RNA Extraction, cDNA Synthesis and Real-Time Reverse Transcription Quantitative Polymerase Chain Reaction (RT-qPCR)

Leaf samples collected from three cultivars under different temperature treatments were ground in liquid nitrogen, and RNA was extracted using the Plant RNA Extraction Kit (TIANGEN Biochemical Technology Co., Beijing, China). Reverse Transcriptase kits (Thermo Fisher Scientific Co. in China, Shanghai, China) were used to synthesize the complementary DNAs (cDNA). RT-qPCR analysis was performed using the GeneRuler DNA Ladder Mix kit (Accurate Biotechnology Co., Changsha, China). The RT-qPCR reaction mixture (10 μL) consisted of 5 μL of SybrGreen qPCR Master Mix, 1 μL of cDNA, 0.2 μL of forward and reserve primers, and 3.6 μL of enzyme-free ddH_2_O. The PCR reaction conditions were as follows: denaturation at 95 ℃ for 30 s, 40 cycles, denaturation at 95 ℃ for 5 min and annealing and extension at 60 ℃ for 30 s. Gene-specific primers were designed using the NCBI Primer-BLAST tool (https://www.ncbi.nlm.nih.gov/tools/primerblast/index.cgi?LINK_LOC=BlastHome, accessed 26 October 2023). All primer sequences are shown in Supplementary Table S3. *GAPDH* served as a housekeeping gene. The relative gene expression was calculated by the formula of 2^−ΔΔCt^ (Supplementary Table S4).

### 3.5. Statistical Analysis

Statistical analyses were performed by one-way ANOVA in combination with Duncan’s test in SPSS version 22.0 (SPSS, Chicago, IL, USA). The means were tested by the least significant difference test (LSD test) at *p* ≤ 0.05. Pearson correlation analysis and figures were drawn using Origin 8.6 software (Origin Lab, Northampton, MA, USA).

## 4. Conclusions

In summary, the differential temperature treatments applied to tea plant varieties LJ, BY, and ZH, significantly influenced chloroplast structure, pigment content, and shoot phenotypes. The results indicated that low temperatures (15 °C) induced more severe chloroplast damage, particularly in BY, while higher temperatures (25–35 °C) resulted in better-developed chloroplasts. Additionally, pigment analysis revealed that chlorophyll content peaked at ambient temperatures but was significantly reduced under low temperatures. BY exhibited resilience to high temperatures, maintaining relatively high chlorophyll content, highlighting its sensitivity, primarily to low temperatures. These observations were further supported by the expression patterns of key pigment synthesis-related genes, which correlated with the changes in chlorophyll and carotenoid contents. This study signifies the critical role of temperature in regulating tea plant pigment content and growth, providing insights for future breeding and cultivation strategies. However, further investigation is necessary to unravel the intricate interactions between temperature, pigment synthesis, and leaf color changes at the molecular level.

## Data Availability

All data are available on request to corresponding author.

## Supplementary Materials

The following supporting information can be downloaded at: https://www.mdpi.com/article/10.3390/ijms251910772/s1.
