# Supplementary material for: Chlorophyll and Carotenoid Metabolism Varies with Growth Temperatures among Tea Genotypes with Different Leaf Colors in Camellia sinensis
Source: Int J Mol Sci. 2024 Oct 7;25(19):10772. doi: 10.3390/ijms251910772 (PMC11477215; doi:10.3390/ijms251910772)
Supplement: Supplementary file 1 [file ijms-25-10772-s001.zip › ijms-3198646-supplementary.pdf]

Supplementary table S1. Correlation analysis of chlorophyll metabolism-related gene expression and chlorophyll content in three cultivars.

**Gene name**	**Index**	**LJ-Correlation**	**LJ-P_value**	**BY-Correlation**	**BY-P_value**	**ZH-Correlation**	**ZH-P_value**
GLURS	chla	0.593	0.320	-0.226	0.774	0.201	0.989
GLURS	chlb	0.680	0.507	-0.284	0.716	0.665	0.933
GLUTR	chla	0.439	0.361	0.979	0.021	0.247	0.753
GLUTR	chlb	0.357	0.643	0.951	0.049	0.544	0.456
GSA-AM	chla	0.569	0.331	-0.105	0.895	0.590	0.410
GSA-AM	chlb	0.545	0.550	-0.219	0.781	-0.115	0.885
PBGS	chla	0.422	0.578	0.813	0.187	0.817	0.183
PBGS	chlb	0.563	0.477	0.740	0.260	0.919	0.081
PBGD	chla	0.372	0.276	0.161	0.839	0.707	0.293
PBGD	chlb	0.377	0.231	0.045	0.955	-0.113	0.887
UROS	chla	0.156	0.844	0.785	0.215	0.821	0.179
UROS	chlb	0.353	0.647	0.707	0.293	0.965	0.035
UROD	chla	0.964	0.036	0.799	0.201	0.992	0.028
UROD	chlb	0.970	0.053	0.732	0.268	0.658	0.342
CPOX	chla	0.795	0.020	0.775	0.225	0.951	0.055
CPOX	chlb	0.669	0.003	0.698	0.302	0.491	0.509
PPOX	chla	0.425	0.575	0.631	0.369	0.989	0.011
PPOX	chlb	0.465	0.535	0.552	0.448	0.797	0.203
MgCh	chla	0.320	0.680	0.846	0.154	0.968	0.032
MgCh	chlb	0.046	0.954	0.783	0.217	0.596	0.404
MgPMT	chla	0.711	0.029	0.895	0.105	0.973	0.027
MgPMT	chlb	0.893	0.011	0.841	0.159	0.649	0.351
MgPEC	chla	0.692	0.308	0.162	0.838	0.676	0.324

MgPEC	chlb	0.887	0.031	0.056	0.944	0.289	0.711
DVR	chla	0.389	0.611	0.588	0.412	0.913	0.049
DVR	chlb	0.611	0.389	0.497	0.503	0.393	0.607
POR	chla	0.118	0.882	0.313	0.687	0.915	0.085
POR	chlb	0.108	0.892	0.212	0.788	0.498	0.502
CHLG	chla	0.245	0.985	-0.624	0.764	0.988	0.001
CHLG	chlb	0.445	0.779	-0.612	0.879	0.668	0.332
CAO	chla	0.236	0.755	0.898	0.102	0.996	0.004
CAO	chlb	0.322	0.555	0.848	0.152	0.671	0.329
NYC1	chla	0.215	0.636	-0.634	0.366	0.493	0.507
NYC1	chlb	0.348	0.652	-0.633	0.367	0.654	0.346
HCAR	chla	0.527	0.473	0.963	0.037	0.076	0.924
HCAR	chlb	0.635	0.365	0.929	0.071	0.577	0.423
CLH1	chla	-0.174	0.773	0.970	0.030	-0.508	0.492
CLH1	chlb	-0.233	0.898	0.990	0.010	0.519	0.806
CLH2	chla	0.201	0.994	-0.063	0.937	0.480	0.520
CLH2	chlb	0.316	0.843	-0.159	0.841	0.740	0.260
SGR	chla	-0.123	0.826	0.866	0.134	0.534	0.466
SGR	chlb	-0.102	0.767	0.819	0.181	0.837	0.163
PAO	chla	-0.118	0.882	0.915	0.085	0.958	0.042
PAO	chlb	-0.239	0.761	0.900	0.100	0.765	0.235
RCCR	chla	0.683	0.417	0.415	0.585	0.990	0.210
RCCR	chlb	0.544	0.563	0.345	0.655	0.165	0.352

Supplementary table S2. Correlation analysis of carotenoids metabolism-related gene expression and carotenoids content.

Gene name	Index	LJ-Correlation	LJ-P_value	BY-Correlation	BY-P_value	ZH-Correlation	ZH-P_value
DXS	carotenoids	-0.252	0.748	0.904	0.096	0.086	0.137
IPI	carotenoids	0.145	0.855	0.161	0.839	-0.107	0.935
GGPPS2	carotenoids	0.321	0.786	-0.145	0.855	0.690	0.031
PSY1	carotenoids	0.249	0.751	0.545	0.455	0.430	0.697
PDS	carotenoids	0.375	0.625	0.705	0.295	-0.255	0.445
Z-ISO	carotenoids	0.638	0.617	-0.328	0.672	0.574	0.262
ZDS	carotenoids	0.238	0.762	0.605	0.040	0.335	0.966
CRTISO	carotenoids	-0.276	0.724	0.575	0.425	0.422	0.782
LCYb1	carotenoids	-0.687	0.313	0.828	0.172	0.207	0.926
LCYb2	carotenoids	0.008	0.221	0.105	0.949	-0.316	0.840
CRTRB2	carotenoids	-0.734	0.266	0.388	0.612	0.282	0.718
BCH	carotenoids	0.896	0.104	0.284	0.716	0.799	0.005
VDE	carotenoids	-0.073	0.927	-0.166	0.993	-0.486	0.142
ZEP	carotenoids	0.666	0.334	0.103	0.972	0.177	0.233
NCED	carotenoids	-0.757	0.243	-0.800	0.200	0.147	0.530
NCED3	carotenoids	0.190	0.810	0.222	0.778	-0.272	0.276
CCD1	carotenoids	0.727	0.273	0.296	0.704	0.007	0.259
lcyE	carotenoids	0.275	0.725	0.505	0.495	0.018	0.382
CYP97A	carotenoids	0.532	0.876	0.348	0.652	0.733	0.267
CYP97C	carotenoids	0.124	0.677	0.616	0.384	-0.283	0.165

Supplementary table S3. Gene-specific primer sequences used in the current study for RT-qPCR experiments.

Gene name	Gene ID	F (5′→3′)	R (5′→3′)
GluRS	CSS0015047	TCCACCAGGGAATCTGAGGA	CATATGAGGCATCAGCCCACT
GluTR/hemA	CSS0011743	ATCAGCAGGGCTAGACTCCC	GCTGATGTTCCTGCCAAAGC
GSAAM/hemL	CSS0020466	GGTCACGCAGATGATGAGGT	CCATGGTAGCAACCCTCGAA
PBGS/ALAD	CSS0022256	ACTTAATAGGCGCCCTCGTC	TCAACACCTGTGGGAGACTT
PBGD/hemC	CSS0024885	TGGCCTTAGCAGGACTCAAAC	GTGCGACAAGAGCCATCCAA
UROS/hemD	CSS0036187	AATTGCTTGGAGCTTCCCCT	GTACCAGCTCCGACTACACC
UROD/hemE	CSS0004968	AAGAGGTGAGGGGTCCAGTT	GTGCGAGTTGTACCCCCTTC
CPOX/hemF	CSS0041680	TGAAACCCCAAAAGATTCTCAGG	TCACACCATTTCTTGAACCGAG
PPOX/hemG	CSS0022330	CACTCCGATGTAGCCCCAAA	AAAGCCTGCCCTGAGTTTCC
MgCh	CSS0016317	AGGAAAGCAGGTGGGAATGAG	CCACGGCCTGTGTCTTTGAG
MgPMT/CHLM	CSS0004907	CATTACCCGCAAAACAAGGCA	GGCACAGCCTCAATCAACCT
MgPEC	CSS0045826	GAACTGGTGCCAGGATGAGAA	TGCAGTTGTCCTGTTCGTCT
DVR	CSS0008645	GGGGAAGGCATTGACACCA	TCCTTCCCATAACTTGGCGTT
POR	CSS0041527	TGACTATCCATCGAAGCGCC	CTCGCATGTCCCCTAGGTTG
CAO	CSS0049616	CATCCGGCCTCCAAGGATAC	ATGGGAGCAAAATCCAGCGA
CHLG	CSS0018345	GCACCAGATAAGGCACCTGT	CCACTCCCCAAACCAATGGA
NYC1	CSS0034731	GGATGCGGGTTGTAAAGGGA	CGGCTTTCAGCCCAATTACG
HCAR	CSS0026751	AAACGGTCCAGACCCATTCC	ATGGACCACAGATTCGAGGC
CLH1	CSS0004684	CTAGCCCTTTCCGGCCATAG	CAACGGGGTCAAGTCCTATT
CLH2	CSS0028283	AAGGAATGTTGCGAACCAGC	AACAAACCTCCTCATGGGCT
SGR	CSS0030812	AGATGCCAAAGCCATGCCAA	GAGTCCGATCCTGACCCGAT
PAO	CSS0024763	GTTCGTATCACGGCTGGTCT	CAAATCGCGCTGAATCGTCA
RCCR	CSS0031276	AGCTTATCCAGAGCAGCCCA	GCGGACCAGAATAGCAGTAGG

DXS	CSS0018056	TGCAAAGGGCTTATGACCAG	AACCATGTGGAAAAGCTCGG
IPI	CSS0026150	TGTGAACCGGGATGAGTTGA	GGAGGGTCCCTTTTTCCACAT
GGPPS2	CSS0007938	AGTCCATGCGCTACTCCCTC	TTCGTAGCTGTTGCGATGTG
PSY1	CSS0002725	AGGCAGGGCTTTCAGATGAG	TGGCGATACAACAACAGCGA
PDS	CSS0024321	GTGGCTGCGTGGAAAGATGA	GTCAAATCGGCTGAACTCCC
Z-ISO	CSS0045415	ATGATGGTGTGCAGTTGTGG	GATTGTGTGAGCCAAGCACC
ZDS	CSS0031670	TTGCTCCAATGCGTGCTTAC	GTAGCACCTTCCATGCTGTCT
CRTISO	CSS0033902	AGGTGTTATTGCCGTTGCCT	TGCCAGTGTCCTTAACCAACC
LCYb1	CSS0017943	TGCGCAACAAGTTTCAGAGG	AGTGGTCATCAACGAACACGA
LCYb2	CSS0031629	TTCAGAGGCAGGGCTTTCTG	GCACCAGACCAGGTAGTGTC
CRTRB2	CSS0044082	GGTCTCTGTTTCGGAGCTGG	TGTCTGAATGGTGGAGCTGG
BCH	CSS0005162	CCTTGTTCCTGGTCTCTGTT	ACTTCCTCAAGTTCCTTGGGTC
VDE	CSS0006565	CAGACGGTGGCTTCTTTACG	ACCATATCCATCCCATGCGTC
ZEP	CSS0031288	CTTGCCTTTGCCTCAGGTTT	AAGGCACCCGATACTGCTTG
NCED	CSS0033791	CCCTTCCATACGCCACCTTC	GCCGTCTTGGGTAGACGATG
NCED3	CSS0015210	TTGCACAGATTGAAGGCATTGT	ACCCAAACATGGCTTGATTTTCC
CCD1	CSS0012422	CGGAGTGCCTGAATGGTGAG	TGCTCTCAGCATTTGCATGTTA
LCYe	CSS0031227	GACTACATCAAGGCCGGTGG	GATCCGGTCCAATCAGTCCA
CYP97A	CSS0044082	GGTCTCTGTTTCGGAGCTGG	TGTCTGAATGGTGGAGCTGG
CYP97C	CSS0037493	AAGGGGCTTGTTGCTGAGG	CGGACTATCAGTGGTAAGGGAG
GAPDH	NA	GGAGCGAGATCCCTCCAAAAT	GGCTGTTGTCATACTTCTCATGG

GluRS - Glutamyl-tRNA synthetase 2. **GluTR/hemA** - Glutamyl-tRNA reductase 3. **GSAAM/hemL** - Glutamate-1-semialdehyde aminotransferase 4. **PBGS/ALAD** - Porphobilinogen synthase 5. **PBGD/hemC** - Porphobilinogen deaminase 6. **UROS/hemD** - Uroporphyrinogen III synthase 7. **UROD/hemE** - Uroporphyrinogen decarboxylase 8. **CPOX/hemF** - Coproporphyrinogen oxidase 9. **PPOX/hemG** - Protoporphyrinogen oxidase 10. **MgCh** - Magnesium chelatase 11. **MgPMT/CHLM** - Magnesium protoporphyrin IX monomethyl ester cyclase 12. **MgPEC** - Magnesium protoporphyrin IX ethyl ester cyclase 13. **DVR** - Dihydropteridine reductase 14. **POR** - Porphobilinogen oxidase 15. **CAO** - Chlorophyllide a oxygenase 16. **CHLG** - Chlorophyll a hydroxylase 17. **NYC1** - Non-yellow color 1 18. **HCAR** - Hydroxycarboxylic acid receptor 19. **CLH1** - Chlorophyllase 1 20. **CLH2** - Chlorophyllase 2 21. **SGR** - Stay green 22. **PAO** - Polyamine oxidase 23. **RCCR** - Red chlorophyll catabolite reductase 24. **DXS** - 1-deoxy-D-xylulose 5-phosphate synthase 25. **IPI** - Isopentenyl diphosphate isomerase 26. **GGPPS2** - Geranylgeranyl

pyrophosphate synthase 2 27. **PSY1** - Phytoene synthase 1 28. **PDS** - Phytoene desaturase 29. **Z-ISO** - Zeta-carotene isomerase 30. **ZDS** - Zeta-carotene desaturase 31. **CRTISO** - Carotenoid isomerase 32. **LCYb1** - Lycopene beta-cyclase 1 33. **LCYb2** - Lycopene beta-cyclase 2 34. **CRTRB2** - Carotenoid isomerase B2 35. **BCH** - Bacteriochlorophyll a hydroxylase 36. **VDE** - Violaxanthin de-epoxidase 37. **ZEP** - Zeaxanthin epoxidase 38. **NCED** - 9-cis-epoxycarotenoid dioxygenase 39. **NCED3** - 9-cis-epoxycarotenoid dioxygenase 3 40. **CCD1** - Carotenoid cleavage dioxygenase 1 41. **LCYe** - Lycopene epsilon-cyclase 42. **CYP97A** - Cytochrome P450 97A 43. **CYP97C** - Cytochrome P450 97C 44. **GAPDH** - Glyceraldehyde-3-phosphate dehydrogenase.

Supplementary table S4. Relative content of chlorophyll metabolism-related and carotenoids metabolism-related gene expression in three cultivars.

**Gene name**	**LJ-15**	**SD**	**LJ-20**	**SD**	**LJ-25**	**SD**	**LJ-35**	**SD**
GluRS	1.627	0.079	1.070	0.124	1.065	0.072	1.015	0.029
GluTR	0.285	0.037	0.972	0.041	1.023	0.160	0.797	0.072
GSA-AM	0.474	0.047	0.958	0.039	0.974	0.062	0.955	0.050
PBGS	0.330	0.040	0.784	0.094	0.980	0.076	2.136	0.019
PBGD	1.405	0.124	1.251	0.125	0.972	0.198	0.829	0.128
UROS	0.897	0.170	0.989	0.048	0.975	0.222	3.531	0.073
UROD	0.454	0.059	0.493	0.094	1.047	0.063	0.857	0.082
CPOX	0.453	0.077	0.818	0.115	0.915	0.167	0.993	0.128
PPOX	0.428	0.060	0.969	0.126	1.053	0.108	1.752	0.223
Mgch	0.253	0.038	1.395	0.077	1.110	0.082	1.061	0.124
MgPMT	0.695	0.111	0.535	0.128	1.049	0.120	1.136	0.073
MgPEC	0.769	0.146	0.618	0.111	1.042	0.108	1.115	0.131
DVR	0.663	0.106	0.404	0.057	1.039	0.199	2.145	0.026
POR	1.135	0.170	0.799	0.124	0.989	0.178	0.596	0.102
CAO	0.876	0.090	0.871	0.096	1.142	0.093	1.933	0.041
CHLG	1.097	0.154	1.120	0.101	0.963	0.072	1.906	0.168
NYC1	0.527	0.053	1.123	0.225	0.898	0.108	1.594	0.143
HCAR	0.456	0.091	0.683	0.109	1.193	0.029	1.650	0.032
CLH1	0.790	0.129	0.525	0.063	1.027	0.136	0.029	0.075
CLH2	1.210	0.092	1.750	0.150	0.983	0.124	4.114	0.032

SGR	0.942	0.183	2.064	0.035	0.981	0.074	2.194	0.049
PAO	0.772	0.093	2.062	0.066	1.030	0.148	1.979	0.194
RCCR	0.369	0.067	1.001	0.200	0.982	0.112	1.158	0.129
DXS	1.008	0.171	1.699	0.097	0.992	0.042	1.307	0.028
IPI	1.279	0.141	1.144	0.160	0.979	0.114	1.722	0.052
GGPPS2	1.226	0.110	0.672	0.067	0.962	0.104	84.298	0.146
PSY1	1.013	0.203	0.880	0.176	0.987	0.237	1.495	0.032
PDS	0.771	0.123	1.000	0.190	1.030	0.198	1.776	0.026
Z-ISO	0.339	0.037	1.348	0.202	1.086	0.143	2.400	0.018
ZDS	1.005	0.181	0.935	0.150	0.995	0.015	1.496	0.029
CRTISO	1.105	0.155	0.984	0.187	0.905	0.012	0.017	0.023
LCYb1	5.784	0.619	0.798	0.128	1.012	0.134	1.384	0.045
LCYb2	2.807	0.067	1.235	0.185	0.945	0.027	1.497	0.039
CRTRB2	2.324	0.130	1.058	0.265	0.943	0.147	0.931	0.021
BCH	0.712	0.092	0.894	0.116	1.045	0.339	0.981	0.044
VDE	1.314	0.112	0.718	0.122	0.976	0.109	1.405	0.029
ZEP	0.636	0.095	0.897	0.126	1.057	0.190	1.275	0.024
NCED	1.657	0.131	1.258	0.151	0.960	0.019	0.695	0.031
NCED3	1.315	0.158	0.638	0.108	0.977	0.141	5.785	0.194
CCD1	0.797	0.190	0.729	0.139	1.027	0.073	1.107	0.021
LCYe	0.965	0.070	0.804	0.088	1.036	0.113	2.047	0.134
CYP97A	1.234	0.084	0.668	0.087	1.043	0.009	1.864	0.074
CYP97C	0.702	0.154	0.874	0.071	1.028	0.151	0.488	0.036

Gene name	BY-15	SD	BY-20	SD	BY-25	SD	BY-35	SD
GluRS	0.450	0.083	1.659	0.028	1.013	0.112	0.709	0.106
GluTR	0.370	0.037	0.608	0.076	1.000	0.044	2.218	0.045
MgPEC	0.542	0.050	0.737	0.045	1.019	0.039	0.671	0.040
PBGS	0.153	0.087	0.623	0.019	0.977	0.091	1.333	0.094
PBGD	0.390	0.060	0.746	0.131	1.002	0.125	0.727	0.127
UROS	0.406	0.046	0.752	0.067	1.026	0.058	1.193	0.059
UROD	0.329	0.099	0.443	0.091	1.056	0.097	1.220	0.103
CPOX	0.300	0.107	0.564	0.152	1.116	0.112	1.176	0.124
PPOX	0.479	0.126	0.501	0.229	1.003	0.124	0.981	0.132
Mgch	0.094	0.009	0.366	0.129	0.918	0.072	1.431	0.073
MgPMT	0.280	0.010	0.477	0.077	0.870	0.126	1.523	0.139
MgPEC	0.133	0.016	0.294	0.137	0.847	0.124	0.516	0.119
DVR	0.215	0.056	0.399	0.027	1.001	0.059	0.920	0.053
POR	0.052	0.010	0.178	0.097	1.008	0.236	0.592	0.229
CAO	0.398	0.094	0.511	0.041	1.004	0.101	1.470	0.094
CHLG	0.674	0.106	0.882	0.175	0.848	0.105	0.881	0.100
NYC1	1.007	0.225	0.515	0.141	1.027	0.220	0.485	0.079
HCAR	0.493	0.083	0.959	0.032	1.016	0.098	1.898	0.100
CLH1	4.292	0.163	5.291	0.074	1.001	0.059	28.414	0.056
CLH2	0.684	0.160	1.049	0.032	1.003	0.148	0.850	0.150
SGR	0.526	0.034	0.491	0.048	1.322	0.034	1.345	0.035
PAO	0.415	0.072	2.002	0.190	1.005	0.060	3.637	0.051
RCCR	0.680	0.108	0.562	0.133	1.032	0.195	0.888	0.113
DXS	0.567	0.116	0.402	0.029	1.002	0.095	0.559	0.098
IPI	1.081	0.171	0.772	0.050	0.879	0.164	0.857	0.165

GGPPS2	0.099	0.058	2.786	0.140	1.000	0.070	0.974	0.067
PSY1	0.652	0.158	0.462	0.032	1.003	0.178	1.489	0.173
PDS	0.081	0.285	0.463	0.027	1.003	0.286	1.341	0.293
Z-ISO	0.684	0.195	0.925	0.020	0.867	0.207	0.490	0.197
ZDS	0.483	0.071	0.703	0.029	1.006	0.154	1.359	0.158
CRTISO	0.535	0.084	0.710	0.024	0.885	0.203	1.393	0.178
LCYb1	0.768	0.116	0.685	0.047	1.032	0.131	1.013	0.126
LCYb2	0.942	0.193	1.093	0.039	1.002	0.194	1.213	0.198
CRTRB2	0.726	0.273	0.701	0.022	1.157	0.272	1.708	0.275
BCH	1.056	0.099	0.749	0.043	1.107	0.113	1.192	0.110
VDE	1.078	0.121	0.741	0.029	1.002	0.125	0.343	0.044
ZEP	1.022	0.120	1.224	0.026	1.004	0.143	1.849	0.140
NCED	3.395	0.151	2.218	0.030	1.002	0.145	2.922	0.140
NCED3	0.365	0.053	1.356	0.176	1.002	0.115	0.977	0.106
CCD1	0.868	0.066	0.760	0.021	1.001	0.136	1.741	0.132
LCYe	0.390	0.089	0.738	0.127	1.004	0.088	1.613	0.124
CYP97A	0.809	0.099	0.667	0.073	1.010	0.085	0.359	0.044
CYP97C	0.681	0.027	0.391	0.037	1.074	0.282	0.322	0.086

Gene name	ZH-15	SD	ZH-20	SD	ZH-25	SD	ZH-35	SD
GluRS	0.490	0.079	1.352	0.029	0.958	0.106	1.791	0.126
GluTR	0.605	0.032	0.852	0.063	1.050	0.037	1.973	0.034
MgPEC	0.246	0.017	0.633	0.052	1.000	0.039	0.271	0.050
PBGS	0.442	0.040	0.171	0.019	1.006	0.091	1.101	0.078
PBGD	0.509	0.039	0.494	0.128	0.906	0.149	0.283	0.084
UROS	0.729	0.190	0.485	0.082	1.139	0.054	1.044	0.050
UROD	0.451	0.054	0.388	0.075	1.037	0.110	0.641	0.094
CPOX	0.384	0.078	0.409	0.130	1.092	0.116	0.534	0.083
PPOX	0.457	0.060	0.302	0.223	1.015	0.124	0.724	0.131
Mgch	0.209	0.038	0.243	0.125	0.944	0.073	0.544	0.085
MgPMT	0.379	0.113	0.384	0.074	0.906	0.130	0.677	0.103
MgPEC	0.575	0.154	0.815	0.138	0.840	0.131	0.905	0.121
DVR	0.228	0.090	0.276	0.022	0.996	0.062	0.324	0.047
POR	0.520	0.197	0.342	0.079	0.951	0.143	0.428	0.091
CAO	0.419	0.219	0.317	0.041	1.031	0.091	0.596	0.099
CHLG	0.530	0.154	0.493	0.068	0.868	0.103	0.722	0.105
NYC1	0.430	0.070	0.606	0.089	1.000	0.212	1.522	0.286
HCAR	1.322	0.093	0.875	0.033	1.064	0.125	6.049	0.113
CLH1	1.865	0.231	1.502	0.075	1.040	0.079	2.162	0.060
CLH2	0.683	0.093	0.676	0.033	1.197	0.157	1.442	0.191
SGR	0.712	0.178	0.546	0.047	1.271	0.031	1.454	0.034
PAO	0.611	0.095	0.576	0.200	0.920	0.057	0.863	0.082
RCCR	0.349	0.067	0.183	0.130	1.060	0.213	0.467	0.191
DXS	0.702	0.190	0.595	0.030	1.047	0.081	0.869	0.140
IPI	1.063	0.141	1.235	0.052	0.929	0.152	0.120	0.155

GGPPS2	0.990	0.094	0.889	0.124	0.963	0.073	0.453	0.051
PSY1	1.090	0.203	0.894	0.032	0.963	0.176	0.485	0.155
PDS	1.781	0.125	1.204	0.026	1.039	0.226	1.544	0.281
Z-ISO	0.558	0.037	0.577	0.018	0.856	0.203	0.912	0.201
ZDS	0.821	0.187	0.760	0.030	0.982	0.160	1.850	0.155
CRTISO	1.190	0.155	0.830	0.023	0.923	0.194	4.618	0.397
LCYb1	0.848	0.074	0.881	0.049	1.003	0.133	1.632	0.131
LCYb2	1.356	0.067	1.332	0.039	1.032	0.233	0.490	0.202
CRTRB2	0.531	0.131	2.028	0.021	1.322	0.272	0.621	0.277
BCH	0.206	0.081	0.052	0.038	1.202	0.121	0.153	0.092
VDE	0.702	0.116	0.683	0.030	0.980	0.114	0.877	0.121
ZEP	0.333	0.083	0.502	0.021	1.069	0.138	0.020	0.103
NCED	0.690	0.132	0.564	0.031	0.900	0.126	1.163	0.145
NCED3	1.954	0.140	1.624	0.172	0.990	0.113	1.310	0.095
CCD1	0.811	0.191	0.232	0.021	0.984	0.133	0.830	0.163
LCYe	0.742	0.070	0.691	0.134	0.984	0.091	1.023	0.092
CYP97A	1.165	0.084	1.228	0.074	0.985	0.076	1.023	0.095
CYP97C	0.207	0.055	0.182	0.036	1.131	0.278	0.708	0.186

SD, Standard Deviation, (each treatment shows the mean, n=3).
